# Comparing the straightness of driving between senior drivers with elevated amyloid burden and those without

**DOI:** 10.1002/alz70857_103449

**Published:** 2025-12-25

**Authors:** Anu M Kumar, Yi Lu Murphy, Amanda Cook Maher, Carol C Persad, Robert A. Koeppe, Bruno Giordani

**Affiliations:** ^1^ University of Michigan, Ann Arbor, MI, USA; ^2^ University of Michigan ‐ Dearborn, Dearborn, MI, USA

## Abstract

**Background:**

Identifying subtle changes in complex daily activities, such as the cognitively demanding task of driving, may reveal insights into the earliest signs of cognitive decline associated with Alzheimer's disease (AD). Specifically, straightness of driving reflects precise lane‐keeping and how well a vehicle maintains a linear path. Deviations from this path may be greater among those early in the AD pathway, potentially providing an ecologically valid marker of early AD risk. This study evaluates the straightness of driving in cognitively normal seniors with and without elevated brain amyloid (Aβ) burden, an early biological marker of AD risk.

**Method:**

Vehicular data was collected during fixed course driving trips of 20 Aβ‐positive and 20 Aβ‐negative consensus‐diagnosed cognitively normal participants (age≥65). Participants drove their own car. PET centiloid scale was used to determine Aβ positivity. Differences in consecutive global positioning system headings were calculated for each participant on each road driven (segments with heading differences <1.8 degrees were considered “relatively straight”, otherwise “deviated”). Independent sample *t*‐tests compared the number, total duration, and driving speed of “relatively straight” segments detected on low and high complexity road types between Aβ‐positive and Aβ‐negative groups.

**Result:**

The number of “relatively straight” driving segments on high‐complexity, moderate to high traffic roads with winding paths were significantly lower among Aβ‐positive drivers than Aβ‐negative (*p* < 0.05). However, the number and duration of “relatively straight” driving segments on single‐lane, low‐traffic roads with straight paths were consistently higher among Aβ‐positive drivers compared to Aβ‐negative (*p*'s <0.05). The driving speed during “relatively straight” segments on certain single‐lane, low‐traffic roads was significantly lower among Aβ‐positive drivers (*p*'s <0.05).

**Conclusion:**

Driving performance of cognitively normal Aβ‐positive older drivers varies depending on the complexity/traffic conditions compared to Aβ‐negative peers. Findings suggest Aβ‐positive drivers perform well on simpler, straight roads (with perhaps more care than Aβ‐negative drivers); however, their driving performance is significantly challenged on complex, winding roads with higher traffic. Further research will include an increased number of participants and diverse road conditions (e.g., night driving, adverse weather scenarios) to gain deeper insights into different factors affecting driving performance in Aβ‐positive seniors.

